# Cellulose and Graphene Based Polyurethane Nanocomposites for FDM 3D Printing: Filament Properties and Printability

**DOI:** 10.3390/polym13050839

**Published:** 2021-03-09

**Authors:** Izaskun Larraza, Julen Vadillo, Tamara Calvo-Correas, Alvaro Tejado, Sheila Olza, Cristina Peña-Rodríguez, Aitor Arbelaiz, Arantxa Eceiza

**Affiliations:** 1 Materials + Technologies’ Research Group (GMT), Department of Chemical and Environmental Engineering, Faculty of Engineering of Gipuzkoa, University of the Basque Country, Plaza Europa 1, 20018 Donostia-San Sebastian, Spain; izaskun.larraza@ehu.eus (I.L.); julen.vadillo@univ-pau.fr (J.V.); tamara.calvo@ehu.eus (T.C.-C.); cristina.pr@ehu.eus (C.P.-R.); 2 IPREM, UMR 5254, E2S UPPA, CNRS, Université de Pau et des Pays de l’Adour, Hélioparc 2, Avenue du Président Pierre Angot, 64000 Pau, France; sheila.olza@univ-pau.fr; 3 TECNALIA, Basque Research and Technology Alliance (BRTA), Area Anardi 5, 20730 Azpeitia, Spain; alvaro.tejado@tecnalia.com; 4 Department of Cellular Biology and Histology, Faculty of Medicine and Odontology, University of the Basque Country, B Sarriena s/n, 48940 Leioa, Spain

**Keywords:** 3D printing, FDM, waterborne polyurethane-urea nanocomposites, nanocomposite filaments

## Abstract

3D printing has exponentially grown in popularity due to the personalization of each printed part it offers, making it extremely beneficial for the very demanding biomedical industry. This technique has been extensively developed and optimized and the advances that now reside in the development of new materials suitable for 3D printing, which may open the door to new applications. Fused deposition modeling (FDM) is the most commonly used 3D printing technique. However, filaments suitable for FDM must meet certain criteria for a successful printing process and thus the optimization of their properties in often necessary. The aim of this work was to prepare a flexible and printable polyurethane filament parting from a biocompatible waterborne polyurethane, which shows potential for biomedical applications. In order to improve filament properties and printability, cellulose nanofibers and graphene were employed to prepare polyurethane based nanocomposites. Prepared nanocomposite filaments showed altered properties which directly impacted their printability. Graphene containing nanocomposites presented sound enough thermal and mechanical properties for a good printing process. Moreover, these filaments were employed in FDM to obtained 3D printed parts, which showed good shape fidelity. Properties exhibited by polyurethane and graphene filaments show potential to be used in biomedical applications.

## 1. Introduction

Additive manufacturing (AM), also known and three-dimensional (3D) printing, has emerged as a new method for material processing. In the last years, its interest and market value have grown exponentially, and this trend is expected to continue in the following years [1,2]. AM printing consists of the layer-by-layer manufacturing of 3D objects modeled by computer aided design (CAD) [3,4]. There are many different additive manufacturing techniques, such as stereolithography (SLA), fused deposition modeling (FDM), selective laser sintering (SLS), and direct ink writing (DIW) [5,6]. Among them, FDM has gained the most popularity, due to its simplicity and cost-effectiveness [5,7]. FDM custom fabrication avoids the use of excess material and the use of molds, and allows the direct preparation of end-use parts [8]. In FDM, a filament is fed to a heated nozzle where it is melted and extruded. The melted material is collected in a plate, layer by layer, following the computer design. Filaments for additive manufacturing must fulfill several requirements. The filament must melt and flow during the manufacturing process, and after the deposition of the layer, material must solidify relatively fast to maintain the printed shape [8]. There are already many commercial filaments on the market, the most popular being based on poly (lactic acid) and acrylonitrile butadiene styrene polymers [4]. However, because of the extent of development of this technique, an increasing interest in the research for new materials for filament with different properties, such as higher flexibility, biocompatibility, electrical conductivity, or antibacterial behavior, is taking place. For example, the use of flexible polyurethane (PU) for 3D printing shows great potential for FDM [9,10,11].

Polyurethanes are block-copolymers consisting in a hard segment (HS), formed by an isocyanate and a chain extender, and a soft segment (SS), formed by a polyol. The HS provides the material with rigidity and strength, while the SS grants flexibility to the polymer. Variating precursors used during the synthesis and ratios between segments, an incredibly large spectrum of polyurethanes with different properties can be obtained [12,13,14]. Moreover, and keeping in mind the current environmental state, a more environmentally friendly alternative to conventional PUs are waterborne polyurethanes (WBPU) and waterborne polyurethane-ureas (WBPUU). WBPU and WBPUU avoid the use of volatile organic compounds (VOC) during their synthesis, from which a water dispersion of polyurethane is obtained. Furthermore, the use of bio-precursors for polyurethanes preparation, such as polyols from soybean oil and fatty acid-derived isocyanates [15,16], has also gained great interest, in order to make these materials more ecofriendly. The possibility to tailor made polyurethane properties for their intended use widens their application to many fields. Moreover, polyurethanes have also shown great biocompatibility and hemocompatibility, which opens the door to the biomedical field [5,10]. The possibility 3D printing offers of producing personalized parts, custom made for the exact shape needed for each case, has made it also a very interesting option for this field [11,17,18]. Flexible medical-grade filaments are being researched, and studies show polyurethanes have potential for this application [11]. However, low rigidity often presents a problem for satisfactory FDM printing and final properties of materials. Materials with high flexibility often show difficulties in the feeding in Bowden extruders [19,20], due to the high friction between the filament and the feeding tube and entanglements of the filaments in the feeding gears. The way to solve this problem could be the addition of nanoreinforcements, such as cellulose and graphene, in order to prepare composite filaments with good printability and interesting properties for different applications. In the literature, there are few works where nanocomposites of PU are used as material for FDM filament [5,7,9,21,22]. The use of nanocomposites in 3D printing is a commonly used option since it can be highly beneficial. The addition of nanoreinforcements can be used both to improve both the printability of materials and the properties of the final printed parts. Nanoreinforcements can be used to modulate filaments and inks rheology [23], to enhance material properties [24,25] or even supply new properties to the materials, such as electrical conductivity [26,27,28].

The incorporation of cellulose nanoentities, such as cellulose nanocrystals (CNC) and cellulose nanofibers (CNF), as nanoreinforcements has gained great interest due to their great specific mechanical and chemical properties, low density, biodegradability, biocompatibility, and large natural availability [29,30]. Fallon et al. [31] studied the addition of CNC to a polyurethane matrix in order to obtain potentially enhanced filaments for 3D printing. They observed that the addition of CNC resulted in filaments with significantly enhanced thermomechanical properties, which could be interesting for FDM. Cellulose is an interesting material for 3D printing. Many cellulose nanoentities and cellulose derivatives can be used in this technique, such as cellulose nanocrystals, cellulose nanofibers, hydroxypropyl methylcellulose, hydroxypropyl cellulose, ethylcellulose, and hydroxypropyl methylcellulose acetate succinate [32,33,34]. Cellulose based materials for 3D printing have shown great mechanical properties due to the reinforcement effect it supplies [35,36,37]. The use of cellulose is of special interest for DIW 3D printing, since it can help modulate the rheological behavior of inks to meet printability requirements [34,38]. Moreover, 3D printed have shown great potential for the pharmaceutical and biomedical industry [32,36]. Hence, the addition of cellulose to a polyurethane matrix could be interesting for its used in 3D printing. 

Other popular nanoreinforcements are carbonaceous nanostructures. Graphite-derived graphene has come up as a great nanoreinforcement option, due to its remarkable specific properties. Graphene shows great mechanical properties, thermal stability, and electrical conductivity, as well as an extremely high aspect ratio, stiffness and barrier properties [39,40]. Chen et al. [5] prepared polyurethane/PLA/graphene oxide filaments and were able to obtain 3D printed parts. Filaments showed good printability and enhanced mechanical properties, and printed parts showed good biocompatibility. Kim et al. [7] used carbon nanotubes to prepare polyurethane based composite filaments and were able to successfully print multiaxial force sensors, facilitating the manufacturing process and further proving the good potential of carbonaceous materials for polyurethane 3D printing. However, there is still much room for further studying and widening the field of PU reinforcement for FDM 3D printing. 

Moreover, both cellulose nanoentities and graphene have shown biocompatible behavior [41,42]. The addition of these nanoreinforcements could potentially improve the properties of a flexible polyurethane filament, making it suitable for FDM and making it a great candidate for the rigorous biomedical field.

In the current work, a WBPUU filament was prepared and characterized. In order to solve printability problems given by extreme flexibility of the filament and obtain printable materials, nanocomposite filaments reinforced with cellulose nanofibers and graphene were also prepared. Composites have been prepared through two different routes, in-situ and ex-situ, and have later been extruded into filaments for FDM printing. Both the effects of the type of nanoreinforcements added and the incorporation method have been studied in the properties of the prepared filaments and their printability. Finally, properties of the printed parts were characterized. The addition of reinforcements is intended to enhance the filament printing process, as well as the final properties of the 3D printed parts. Potentially, these improved properties could make these flexible materials a great possibility for biomedical applications where less rigid materials are needed. 

## 2. Materials and Methods

The renewable sourced difunctional polyol used in the synthesis of the waterborne polyurethane was a Priplast 3192^®^ (Mw = 2000 g mol^−1^, Croda (Snaith, UK)) and the isocyanate was an isophorone diisocyante (IPDI, DESMODOUR I), kindly supplied from Covestro (Leverkusen, Germany). 2,2-Bis(hydroxymethyl)propionic acid (DMPA), provided by Aldrich (St. Louis, MI, USA) was used as internal emulsifier and ethylene diamine (EDA), provided by Fluka (Buchs, Switzerland), was used as chain extender. To neutralize the carboxylic groups of the emulsifier, triethylamine (TEA, Fluka) was employed and 0.037 wt% of dibutyltin dilaurate (DBTDL), provided from Aldrich, was used as catalyst. 

For cellulose nanofiber preparation, standard bleached hardwood kraft pulp (bHKP), supplied by local paper mill, was used. In order to optimize the compatibility between the polymer and cellulose, the carboxylation of cellulose OH groups was done. During the treatment process of the cellulose, sodium metaperiodate (NaIO_4_), sodium chloride (NaCl), hydrogen peroxide (H_2_O_2_), sodium hydroxide (NaOH) and sodium chlorite (NaClO_2_) were employed, all were purchased from Scharlab (Barcelona, Spain).

For the obtaining of graphene, graphite flakes (Gr) purchased from Aldrich were employed. Sulfuric acid (H_2_SO_4_, 96%), sodium nitrate (NaNO_3_, 99%), potassium permanganate (KMnO_4_, 99%), hydrogen peroxide (H_2_O_2_, 30% *w*/*v*) and hydrochloric acid (HCl, 37%) used during this process were supplied by Panreac. Finally, *Salvia officinalis L*. was purchased at a local herbalist. 

### 2.1. Synthesis of the Waterborne Polyurethane-Urea

A waterborne polyurethane-urea, with a molar ratio of polyol/DMPA/IPDI/EDA of 1/1.1/3.5/0.6, was synthesized using a two-step polymerization process. The procedure was carried out in a 250 mL four-necked flask equipped with a mechanical stirrer, thermometer and nitrogen inlet within a thermostatic bath. In the first step the prepolymer was synthesized, combining the polyol, the diisocianate and the catalyst and letting it react for 5 h at 100 °C under constant mechanical stirring. Afterwards, the reaction was cooled down to 50 °C and the DMPA and the neutralizing TEA were added dissolved in acetone and let to react for 1 more hour. For the second step of the synthesis, the reaction was cooled down to room temperature, and the phase inversion step was carried out by dropwise addition of deionized water under vigorous stirring. Finally, the chain extender was added to the reaction at 35 °C and left to react for 2 h. An aqueous dispersion of WBPUU with a solid content of 33 wt% was obtained.

### 2.2. Obtaining of CNF, Carboxylated CNF and Graphene

For the preparation of CNF, first bHKP sheets were cut in pieces and left to swell on water for 24 h. Then, using mechanical agitation, the mixture was dispersed until homogeneity was achieved, with no visible agglomerations. Finally, the suspension was passed through a Masuko Supermass Colloider (MKZA10-15J, Masuko Sangyo Co., Kawaguchi, Japan), until no microstructures were present, and thus mechanically achieved cellulose nanofibers were obtained. The isolated nanofibers were denominated as CNF0.

In order to functionalize CNF and obtain carboxylated cellulose nanofibers, a sequential periodate-chlorite oxidation treatment was carried out parting from the pulp suspension prior to the Masuko [43,44]. Two sequential reactions were carried out during this process. In the first reaction, the cellulose fiber water suspension was mixed with sodium metaperiodate and NaCl, and was left to react under total darkness for 2 h. In the second reaction, the resulting suspension was mixed with NaClO_2_, NaCl, and H_2_O_2_, and the mixture was once again left to react for 2 h. The pH during this time needed to be maintained between 4.2 and 4.5, in order to do so, NaOH was used. Finally, the reaction mixture was filtered and the obtained dicarboxylated cellulose fibers washed repeatedly. Lastly, in order to obtain nanostructures, the prepared carboxylic cellulose was disintegrated in the Masuko Supermass Colloider until no microstructures were observed under the optical microscope. The obtained carboxylated cellulose nanofibers were denominated CNF1.

Graphene (G) was obtained by thermal reduction of graphene oxide (GO). First graphene oxide was produced using Hummer’s method parting from graphite flakes. Briefly, a mixture of graphite flakes, NaNO_3_ and H_2_SO_4_ were reacted for 30 min at 0 °C, after which KMnO_4_ was added, and the new mixture was kept under agitation for 2 h. The reaction was heated to 35 °C for 30 min and water was later added, drop by drop. It was kept under agitation at 98 °C during the selected oxidation time, 30 min, and then H_2_O_2_ was added and the reaction was left to cool to room temperature. Finally, water was added. The mixture was centrifuged, first with 5% HCl and later with deionized water, until neutral pH was achieved. At last, graphite oxide was exfoliated using sonication and the largest flakes of GO were eliminated via centrifugation. 

In order to get rid of functional groups in the surface of the graphene oxide structure and obtain graphene, GO was thermally treated, keeping it a 500 °C for 30 min. Afterwards, a partially reduced graphene oxide, with a much-reduced amount of oxygenated groups in the surface, was achieved.

### 2.3. Composite Preparation

According to previous works [44,45], it was observed that a 3 wt% content of reinforcement resulted in a great reinforcement effect, thus, composites containing this amount of nanoreinforcements content (CNF0, CNF1 and G) were prepared by two different incorporation routes, ex-situ, by sonication of the WBPUU and the reinforcement, and in-situ, during the synthesis of the WBPUU. 

First, a good water dispersion of the reinforcements was ensured. CNF0 and CNF1, already in aqueous dispersion (6 mg mL^−1^), were sonicated for 1 h in a sonication bath. In the case of G, in order to enhance water dispersability the use of a surfactant was necessary. Plant extracts have shown great potential as natural surfactants, especially when working with graphene due to the π–π interactions. In the current study, *Salvia officinalis* (E) was used with a weight ratio of G/E equal to 2/1, as proposed by Gonzalez et al. [46]. G was sonicated in the water/*salvia* (5 mg mL^−1^) solution for 5 h to ensure good dispersion. 

In the case of ex-situ composites, nanoreinforcement dispersions were added to the WBPUU dispersion, and the new mixtures were further sonicated for 1 h. For in-situ composites, the aqueous dispersions of the reinforcements were added drop by drop to the synthesis during the phase inversion step. Each preparation, as well as the WBPUU, was poured in a Teflon mold and left to dry at room temperature for 7 days, followed by 3 days under vacuum to remove any possible water trace. Thus, films were obtained for the neat WBPUU, the ex-situ composites (3CNF0_EX_, 3CNF1_EX_ and 3G_EX_) and in-situ composites (3CNF0_IN_, 3CNF1_IN_ and 3G_IN_).

### 2.4. Extrusion Process

A twin screw extruder, HAAKE MiniLab extruder (Thermo Fisher Scientific (Waltham, MA, USA)), with a 1.75 mm nozzle was used to obtain composite filaments. As pellets, pieces of 5 × 5 mm^2^ size cut from films were used. Extrusion was done with a feeding flow of 0.3 g every 25 s at 50 rpm. Temperature was optimized for extrusion process of the systems. WBPUU and composites containing graphene were extruded at 160 °C. WBPUU/CNF composites were processed at a higher temperature, ex-situ and in-situ composites were extruded at 180 °C and 190 °C, respectively. The filament was extruded directly onto a conveyer belt where it cooled down and completely solidified. Prepared filaments of neat WBPUU and composites were named FWBPUU, FCNF0_EX_, FCNF0_IN_, FCNF1_EX_, FCNF1_IN_, F3G_EX_, and F3G_IN_.

### 2.5. Fused Deposition 3D Printing

**Figure 1:**
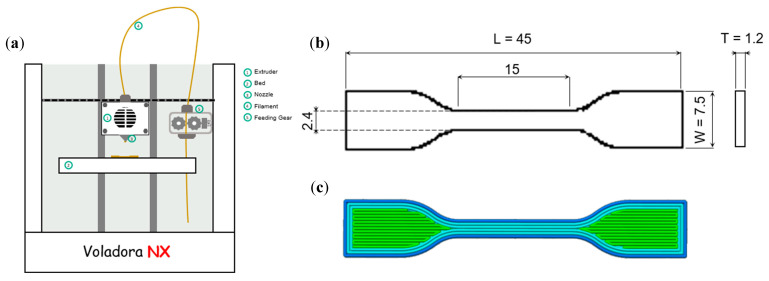
(**a**) Schematic model of the Tumaker Voladora NX 3D printer, (**b**) dimensions of imported dog-bone design and (**c**) computer aided design and infill pattern.

3D printing was carried out using the fused deposition fabrication method, with a Tumaker Voladora NX printer (Figure 1a) paired with a Simplify3D software. Printing parameters were optimized for the prepared systems (Table 1). Designed models for 3D printing were dog-bone specimens (Figure 1b). Infill percentage was set at 100% and a rectilinear printing pattern with an angle of 0° was chosen (Figure 1c).

Whereas 3D printing was only possible for filaments containing graphene, printed parts for F3G_EX_ and F3G_IN_ filaments were named 3D3G_EX_ and 3D3G_IN_, respectively.

### 2.6. Characterization

#### Biocompatibility Analyses

In order to analyze in vitro biocompatibility of the synthesized WBPUU, cytotoxicity and cell adhesion (Live/Dead assay) analysis were performed. For both tests, a WBPUU film prepared by solvent casting was used. Cytotoxicity was evaluated following ISO 10993-5:2009 standard protocol and by PrestoBlue^®^ (Invitrogen), a resazurin-based solution that functions as a colorimetric cell viability indicator. Briefly, murine fibroblasts (L929 cells) were seeded into 96-well plates at a density of 4 × 10^3^ cells/well in 100 μL of complete culture medium (Dulbecco’s modified Eagle’s medium (DMEM) supplemented with sodium pyruvate1 mM, 1% of non-essential amino acids, 1% penicillin-streptomycin and fetal bovine serum 10%). After 24 h, the medium was replaced with 100 μL of negative control (fresh complete culture medium), positive control (DMSO, 10% in complete culture medium) or biomaterial’s extractive media and a 10% of PrestoBlue^®^ was added. The optical density (OD) was measured at 570 and 600 nm in a spectrophotometer (Synergy HT spectrophotometer, Biotek, Winooski, VT, USA)) at different time points (0, 24, 48 and 72 h). The viability of the cells was calculated from equation 1. All assays were conducted in triplicate and average values and their standard deviations were calculated. For the in vitro biocompatibility assay, a two-way analysis of variance (ANOVA) followed by Bonferroni post-test was performed using GraphPad Prism software (San Diego, CA, USA). The results were expressed as mean ± SD and values of *p* < 0.05 were considered statistically significant with respect to the positive control. All assays were conducted in triplicate.

(1) $$\mathrm{Viability}\;\left(\%\right)=\frac{{\mathrm{Abs}}_{\mathrm{sample}}}{{\mathrm{Abs}}_{\mathrm{negative}\;\mathrm{control}}}\;\times \;100$$

where Abs_sample_ is the absorbance of the sample cells cultured in biomaterial’s extractive media and Abs_negative_ control is the absorbance of the negative control.

The adhesion of the L929 cells on the surface of the materials was studied by performing Live/Dead assay. WBPUU samples of 0.5 cm^2^ were prepared and sterilized under ultra-violet light for 30 min prior to analysis. The material was placed in 24-well ultra-low attachment plate and incubated at 37 °C for 24 h in 500 μL of a complete culture medium. After that, the medium was removed from the wells and L929 were seeded on the surface of the material at a density of 5 × 10^4^ cells in 20 μL of complete culture medium and were incubated for 2 h at 37 °C to enhance cell adhesion onto the materials surface. To maintain a hydrated environment, 500 μL of PBS were added in the adjacent well plates. After the period of adhesion, 500 μL of complete culture medium were added to each sample. Fluorescent images were obtained in a confocal microscope (Olympus LV500, Shinjuku, Japan) after 3 and 7 days. The medium was removed and the samples were rinsed twice with PBS and later dyed with Calcein AM 4μm and propidium iodide 5 μM in 1 mL of PBS. Finally, samples were incubated for 20 min at 37 °C in the dark. Live cells were observed thanks to the green fluorescence of the Calcein AM (λex/λem: 495/515) and dead cells due to the red fluorescence of the propidium iodide (λex/λem: 535/617 nm).

### 2.7. Fourier Transform Infrared Spectroscopy (FTIR)

The characteristic functional groups of the used nanoreinforcements, neat WBPUU and the composite filaments were analyzed by Fourier transform infrared spectroscopy using a Nicolet Nexus (Thermofisher Scientific, Waltham, MA, USA) spectrometer provided with a MKII Golden Gate accessory (Specac) with a diamond crystal at a nominal incidence angle of 45° and ZnSe lens. Spectra were recorded in attenuated total reflection (ATR) mode between 4000 and 650 cm^−1^ averaging 32 scans with a resolution of 4 cm^−1^.

### 2.8. Thermogravimetric Analysis (TGA)

The thermal stability of the used nanoreinforcements, the WBPUU and the composites were determined by thermogravimetric analysis. (TGA) was performed in a TGA/STDA 851 (Mettler Toledo, Columbus, OH, USA) equipment. The samples, between 5 and 10 mg, were heated from 30 to 700 °C in a nitrogen atmosphere at a scanning rate of 10 °C min^−1^.

### 2.9. Dynamic Mechanical Analysis (DMA)

Dynamic mechanical analysis was carried out to study the thermomechanical behavior of the filaments and printed pieces, using an Eplexor 100 N analyzer Gabo (Selb, Germany) equipment. Measurements were carried out in tensile mode in a temperature range from −100 to 180 °C at a scanning rate of 2 °C min^−1^. The initial strain was established as 0.05% and the operating frequency was fixed at 1 Hz.

### 2.10. Mechanical Testing

Mechanical tests were performed in an Instron 5967 testing machine (Instron, Norwood, MA, USA) provided with a 500 N load cell and pneumatic grips to hold the samples. Filaments with a 1.75 mm diameter were tested at a crosshead speed of 20 mm min^−1^ at room temperature with a distance between clamps of 10 mm. For 3D printed pieces, dog-bone specimens were used, with a gauge length, width and thickness of 50, 3.25 and 1.3 mm, respectively. Tensile modulus (E), stress at yield (σ_y_), stress at break (σ_b_) and elongation at break (ε_b_) were determined from stress-strain curves of five specimens of each series. In order to consider the significance of the results ANOVA was carried out. ANOVA was conducted with OriginPro8 (Origin Lab), using the Turkey’s test at a significant level of 0.05.

### 2.11. Scanning Electron Microscopy (SEM)

The morphology of the 3D printed pieces was analyzed via electron scanning microscopy by a Field Emission Gun Scanning Electron Microscopy (FEG-SEM) Hitachi S-4800N (Hitachi High-Tech, Tokyo, Japan), at a voltage of 5 kV. Prior to the test, sample were first frozen in liquid nitrogen and a cryofracture of the cross-section was done. Samples were sputter coated with a thin layer of gold (~10 nm) in an Emitech K550X ion sputter. The morphology of the 3D printed pieces and layer adhesion was observed in SEM images. 

## 3. Results and Discussion

### 3.1. Characterization of the Different Nanoreinforcements

**Figure 2:**
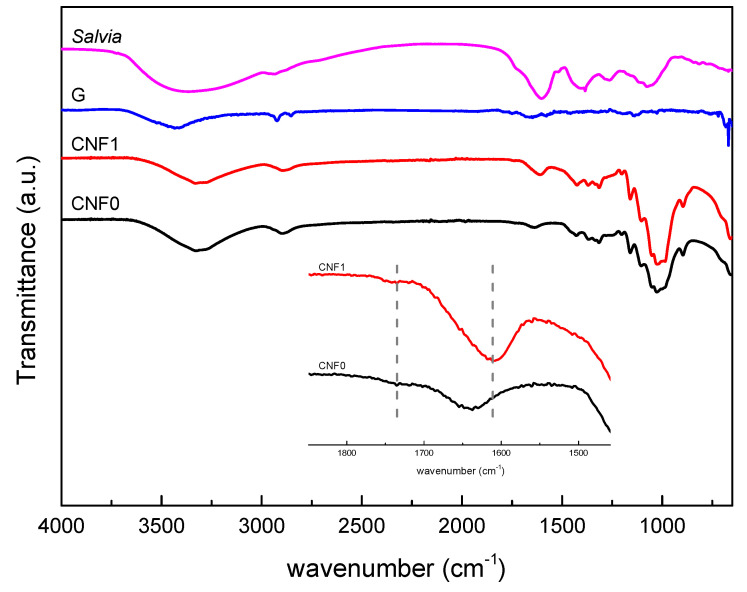
FTIR spectra of CNF0, CNF1, graphene and *salvia* extract.

The characteristic functional groups of the nanoreinforcements were studied by Fourier transform infrared spectroscopy, the obtained spectra are shown in Figure 2. Regarding cellulose nanofibers both spectra showed the characteristic bands of cellulose, with at band at 3330 cm^−1^, corresponding to the stretching vibration of the O–H groups, bands at 2900–2800 cm^−1^ and 1429 cm^−1^, attributed to C–H stretching vibration and symmetric bending of C–H_2,_ respectively, a band associated with the absorbed water at 1635 cm^−1^, bands corresponding to C–O–C asymmetric stretching in β-glycosidic linkages at 1160 and 897 cm^−1^, and a band at 1031 cm^−1^ from the C–O stretching at C6 [47,48,49]. When analyzing the carbonyl stretching vibration region, the effect of the carboxylation process can be observed. Both systems show a small shoulder at 1735 cm^−1^, corresponding to protonated carboxyl groups [50]. This band is slightly more pronounced in the spectra of the treated system. Moreover, the major difference between both spectra is the band located at 1611 cm^−1^ for CNF1, associated with unprotonated carboxyl groups [50], which does not appear for CNF0 (Figure 2 inset). 

Regarding G spectrum, a very low intensity spectrum was obtained. Most significant bands appeared at 3434 and 1647 cm^−1^, corresponding to O–H stretching and bending vibrations and at 2929 and 2854 cm^−1^ related to C–H stretching vibration [51,52,53]. The presence of these bands suggested that only a partial reduction was achieved during the graphene obtaining process, and some functional groups remain in the G structure. This partial reduction was already observed in a previous study [45]. *Salvia* spectrum can also be seen on Figure 2. *Salvia* extract spectrum shows two main characteristic bands, a wide band between 3700–3000 cm^−1^, attributed to the hydroxyl groups in the *salvia* structure and a wide band between 1800–1500 cm^−1^, related to an overlapping of the carboxylic group band and the C=C aromatic rings [54].

**Figure 3:**
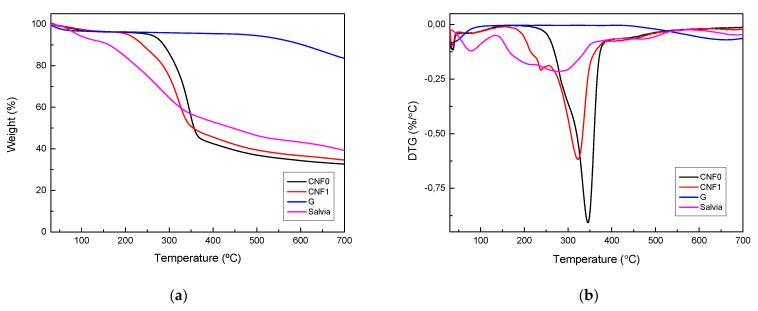
(**a**) TGA and (**b**) DTG curves of CNF0, CNF1, graphene and *salvia* extract.

TGA were performed for all three nanoreinforcements, as well as *Salvia* extract (Figure 3). For G a very reduced weight loss was observed throughout the thermal scan, showing a high thermal stability, characteristic of purely carbonaceous structures. A small drop at low temperatures can be observed, related to the evaporation of absorbed moisture. Moreover, at high temperatures, around 660 °C, a slight weight loss can be seen, which can be attributed to the remaining oxygen containing groups in the structure [55], which is in agreement with FTIR results.

Regarding CNF samples, TGA and DTG curves show slight differences for degradation process of each CNF system. For CNF0, a single peak is observed in DTG curve, related to the degradation of cellulose, going from 250 to 390 °C and the maximum degradation temperature located at 346 °C [56]. In the case of CNF1, the onset degradation temperature (205 °C) and maximum degradation temperature (322 °C) are located at lower temperatures than CNF0 system. Such a reduction of the degradation temperature could be due to damaging of the crystals during the carboxylation processs [56]. Moreover, CNF1 DTG curve shows also an intense shoulder at 230 °C, which can be attributed to their nanometric size and the larger amount of free ends present [57]. Regarding the *salvia* extract, it shows an initial loss around 100 °C, related to the evaporation of humidity. After that, a constant weight loss takes places, most of it between 150 and 400 °C, where the decomposition of the polysaccharides takes place [58].

### 3.2. Biocompatibility of WBPUU

**Figure 4:**
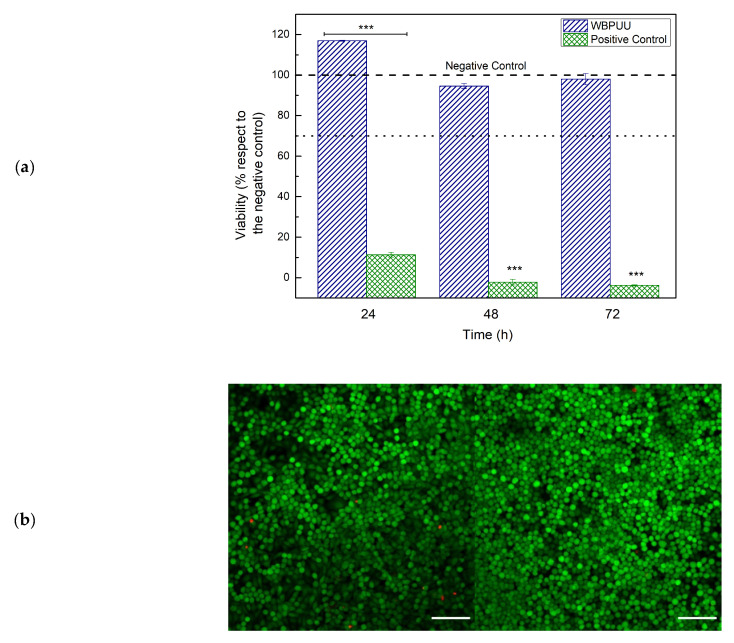
(**a**) Viability of L929 murine fibroblast cells on WBPUU as function of incubation time. *Dotted line represents the maximum value of viability given by the negative control and dashed line represents the limit of acceptance stablished by ISO 10993-5:2009 (70% of the value of the negative control); * *p* < 0.05. (**b**) Adhesion and viability (Live/Dead assay) of L929 cells on WBPUU surface after 3 (**left**) and 7 (**right**) days. Scale bar on confocal microscopy images represents 100 μm.

In order to analyze biocompatibility of the synthesized WBPUU and evaluate its possible use in the biomedical field, cytotoxicity analyses were carried out with L929 murine fibroblast cells. Figure 4a shows short term cell viability with respect to the negative control, after 24 and 48 h. The viability results obtained for WBPUU showed higher values than the acceptable minimum establishes by the ISO 10993-5 (70% of the negative control value), proving a good non-toxic behavior.

Moreover, cell adhesion and proliferation assays were also done. Obtained images are shown in Figure 4b. After 3 days, it can be seen that cells are viable (cells in green) and they show a homogenous distribution throughout the surface of the WBPUU. The amount of dead cells is extremely low and the cellular density is very high, leaving very few uncolonized zones. This trend is maintained a week after the seeding, where the density of the viable cells has significantly increased. The cell density is so high, that cells have begun to grow on top of each other. The material proved to be a perfect environment for the adhesion and growth of the cells. 

### 3.3. Characterization of Filaments

**Figure 5:**
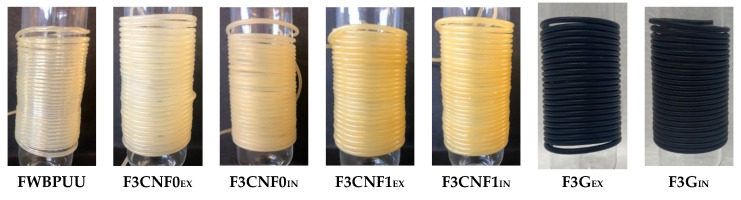
Photographs of the prepared in-situ and ex-situ filaments.

Figure 5 shows pictures of the extruded filaments. FWBPUU has a transparent aspect, which changes with the addition of the nanoreinforcements. WBPUU/CNF0 filaments show a slightly more yellow tonality, more noticeable for the composite prepared in-situ. This same yellow tonality is also seen in WBPUU/CNF1 filaments, in the case of these materials this yellow-brownish color is more intense. This change in the pigmentation of the material could be due to the cellulose beginning to degrade during the extrusion process, due to the thermoxidative conditions used. The faster degradation observed for the modified cellulose would agree with the darker tonality in the composites containing CNF1. Regarding filaments reinforced with graphene, both filaments, F3G_EX_ and F3G_IN_, are completely black. In this case, the black color of graphene overtakes the color of the matrix. It is also worth noting that the extrusion of composites containing cellulose was more difficult to control than graphene-based systems, causing a higher variation of filament diameter. This effect was more noticeable for composites prepared in-situ. Thus, a higher extrusion temperature was needed for these systems than for ex-situ ones. On the other hand, the extrusion of graphene-based material was more constant and, thus, filaments with a more homogeneous diameter were obtained. 

**Figure 6:**
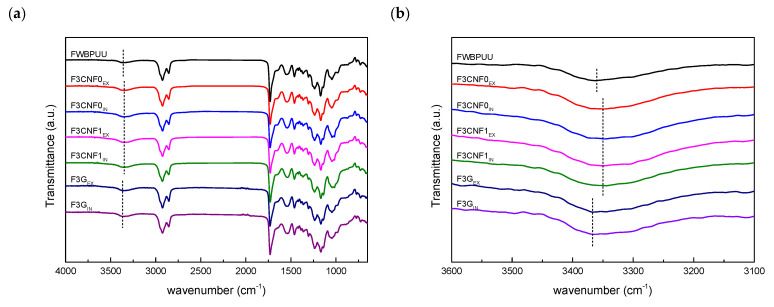
(**a**) FTIR spectra of matrix and composite filaments and (**b**) zoomed FTIR spectra for the 3600–3100 cm^−1^ region.

Fourier transform infrared spectroscopy analyses were carried out in order to assess possible interactions taken place between the WBPUU matrix and the nanoreinforcements used. FTIR spectra of WBPUU filament and composite filaments are shown in Figure 6.

FWBPUU spectrum shows characteristic bands of polyurethanes: an absorption band corresponding to H-bonding of polyurethanes’ N–H groups at 3360 cm^−1^, a band attributed to carbonyl vibration of the polyol and urethane groups at 1730 cm^−1^, bands at 1645 cm^−1^ and 1545 cm^−1^, assigned to carbonyl stretching vibration of the urea groups, and C–N stretching vibration and N–H bending of urethane and urea groups, respectively, and bands in the 1250–1000 cm^−1^ region corresponding to C–O stretching vibrations [59,60,61].

When analyzing composite spectra, it can be observed that all systems show similar spectra with only slight differences in the N–H band. For composites containing cellulose nanofibers, this band displaced to lower wavenumbers, indicating H-bonding taking place between the polyurethanes and cellulose molecules. This change took place for all WBPUU/CNF composites. However, no differences were observed based on the type of cellulose employed or the incorporation route. Composites containing G presented a change in wavenumber, however, in this case the band displaced to slightly higher wavenumber values. This change could be due to an overlapping of the matrix N–H band with the O–H band of G and E. 

**Figure 7:**
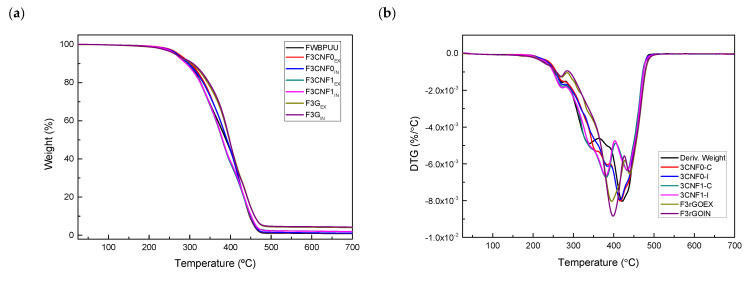
(**a**) TGA and (**b**) DTG curves for matrix and composite filaments.

In order to study the thermal stability of the filaments, thermogravimetric analysis was carried out. Thermal degradations of WBPUU and composite filaments are shown in Figure 7. FWBPUU thermal degradation can be separated in two steps, the degradation of the HS and the SS, which can be seen in the DTG curves in Figure 7b. The degradation of the shorter chains in the HS takes place at lower temperatures. A peak at 340 °C can be observed, preceded by a shoulder at 272 °C, these degradation regions belong to the degradation of urethane and urea groups, respectively [62]. At higher temperatures, the second step of the degradation process of WBPUU takes place. A peak at 420 °C for the degradation of the SS can be observed [63]. 

When studying thermal stability of WBPUU/CNF composites, small differences can be observed. Filaments based on CNF0 show slightly higher degradation temperature than FWBPUU one. This slight stability improvement could be due to the stabilization of urea and urethane groups from interactions taking place between matrix and CNF0 [44,45], agreeing with FTIR analyses. For WBPUU/CNF1 filaments this improvement is not observed, probably due to the lower degradation temperatures observed for carboxylated cellulose nanofibers. 

For WBPUU/G composite filaments, an enhancement on the thermal stability of the materials with the addition of graphene can be observed. In the case of filaments containing graphene both the SS and HS degradation temperatures are displaced to higher temperatures, showing an enhancement of the thermal stability with the addition of graphene [64,65].

The mechanical properties of the filaments were also studied to observe possible changes taken place with the addition of CNF and G (Table 2). The significance of the changes taken place in the nanocomposite films was analyzed by statistical analysis and are shown in Table 2. ANOVA analyses were carried out for each parameter and values with same letter represent no significance between values. The addition of nanoreinforcements significantly altered the mechanical behavior of the materials in at least one of the studied parameters. Most of the systems showed significantly altered properties in all parameters. Regarding composites containing CNF, it can be observed that the addition of CNF resulted in an increase in Young modulus values, however the ex-situ prepared composites show a higher enhancement than in-situ prepared composites. Young modulus for ex-situ composites increases up to a 383% in the case of F3CNF0_EX_ and a 245% for F3CNF1_EX_. F3CNF0_EX_ shows also a significant enhancement is the stress at break value. This mechanical enhancement with the addition of cellulose nanofibers shows the reinforcement effect supply by cellulose nanoentities, and is in agreement with reports in literature [44,66]. However, in-situ composites showed similar or slightly lower values than neat polymer filament, and F3CNF1_IN_ even small deteriorations. All WBPUU/CNF composites suffered a pronounce drop in the strain at break values, which could probably be ought to small degradation taken place during the high temperature extrusion process, as suggested by changes in material color, which would also explain the poor reinforcement effect supplied by the incorporation of CNF compared to previously studied composites films [44]. 

WBPUU/G composites show a very different behavior. Both composites, prepared via ex-situ and in-situ, show improved mechanical properties. In this case significantly enhanced Young modulus and stress at break values are obtained for G containing composites, which is in agreement with reported literature for polyurethanes reinforced with carbonaceous nanostructures [67,68]. Moreover, besides a clear improvement in the yield and break stresses of materials, good elasticity properties are maintained. Strain at break values shown by WBPUU/G filaments are similar to those shown by FWBPUU, even slightly higher. The good mechanical properties observed for composites containing graphene could be due to the effect of the addition of *salvia* as well as graphene, as seen in previous studies [45]. The lower temperature needed for the satisfactory extrusion process in the case of WBPUU/G composites resulted in less degraded filaments. 

**Figure 8:**
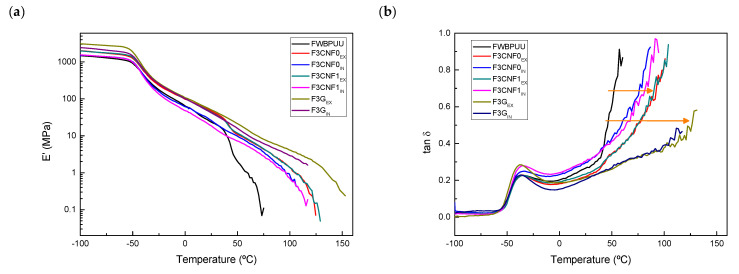
(**a**) Storage modulus and (**b**) tan δ curves of matrix and composite filaments.

Dynamic-mechanical analyses were carried out to analyze the thermo-mechanical behavior of the filaments. When analyzing the DMA results, the reinforcement effect of CNF and G can be clearly seen (Figure 8). 

Until glass transition temperature (T_g_) is reached, which can be associated with the maximum of tan δ curve, systems show a constant modulus, and at a same temperature, the modulus value varies with the type of reinforcement and the incorporation method used. Composites reinforced with graphene show a significantly higher storage modulus than composites reinforced with CNF. This higher storage modulus could be due to high modulus and specific surface area of graphene [69]. Composites prepared ex-situ showed also higher values than in-situ composites. The addition of nanoreinforcements resulted in materials with higher modulus values than unreinforced system and highly enhanced thermomechanical stability. The FWBPUU storage modulus begins dropping at around 40 °C, whereas for the composite filaments it is delayed about 50 °C. At this same temperature, it can be seen that tan δ curves tend to infinity. The mentioned delay can also be seen in tan δ curves, where the higher stability increase in graphene containing composites can be clearly seen (signaled with arrows in Figure 8b). The reduced mobility of the chains, due to the H-bondings observed by FTIR and the reinforcement effect of G and CNF increased material stiffness and thus storage modulus [70].

When studying the effect of the type of nanoreinforcements used, no relevant changes were observed for WBPUU/CNF1 composites compared to WBPUU/CNF0 composites. Nanocomposites containing graphene showed a more pronounced reinforcement effect, F3G_EX_ and F3G_IN_ show higher modulus values and are able to maintain the structural integrity of the material at higher temperatures [65,70].

### 3.4. Characterization of 3D Printed Parts

**Figure 9:**
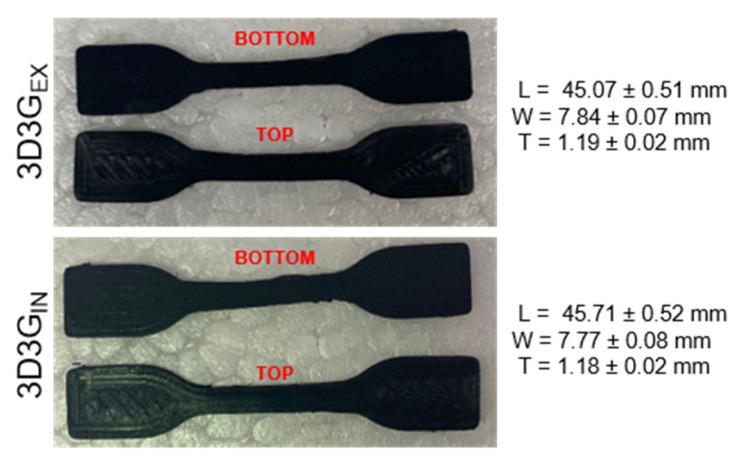
Photographs of 3D printed parts based on graphene systems.

After characterization of the prepared filaments, dog bone specimens were obtained by FDM 3D printing (Figure 9). Variating extrusion temperature, collecting bed temperature and printing velocity, the printing process was optimized for these WBPUU based filaments. It was observed that the addition of nanoreinforcements, as well as the type of nanoreinforcements, severely affected the printing capacity of the material with the Tumaker Voladora NX printer. FWBPUU, FCNF0_EX_, FCNF0_IN_, FCNF1_EX_ and FCNF1_IN_ presented different problems during printing process. Filament stiffness in these cases was not enough for a good feeding of the material, making the filament bend around feeding gear instead of pushing it to extruder. Moreover, for filaments with better feeding capacity and after optimization, the extruding flow was too slow and did not allowed a continuous printing without nozzle obstruction, this could be attributed to low pressure on the nozzle due to the extreme flexibility of the filaments and to the high viscosity of these filaments due to the interactions observed by FTIR [8,71].

In the case of filaments containing graphene, the better mechanical behavior shown by these filaments allowed both filaments to show a good printing capacity, with good flow and adhesion between layers. The lower flexibility shown by these filaments resulted in a better printability of the systems. A quite loyal reproduction of the shape and size of the imported model was obtained in the printed parts. (Figure 9).

**Figure 10:**
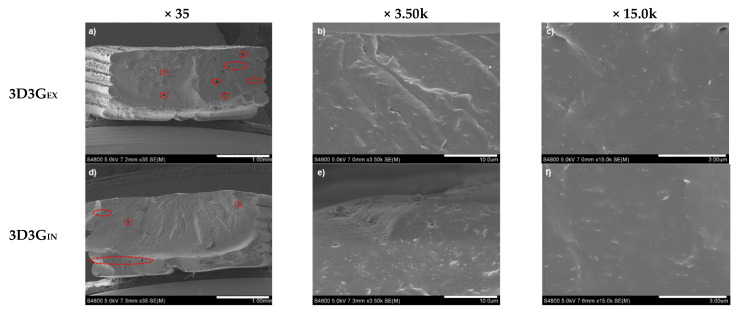
SEM images for 3D3G_EX_ with a magnification of (**a**) 35, (**b**) 3.5k and (**c**) 15.0k; and for 3D3G_IN_ with a magnification of (**d**) 35, (**e**) 3.5k and (**f**) 15.0k.

In order to further analyze the morphology of the printed material, SEM analyses were carried out, obtained images are shown in Figure 10. Both systems presented similar morphologies. Figure 10a,d show low magnification images of 3D3G_EX_ and 3D3G_IN_ systems, respectively, where imperfections formed during the printing process can be observed. Though both printed parts show, in general, a compact interior, smalls holes (circled in red) can be observed throughout the transversal cut. Most of these holes seem to align with the junction of the layers, signaling to some adhesion problems between layers. Figure 10b,c,e and fshow more magnified images of the materials. A good dispersion of the nanoreinforcements can be deduced from these images, since no apparent agglomerations can be observed. 

**Figure 11:**
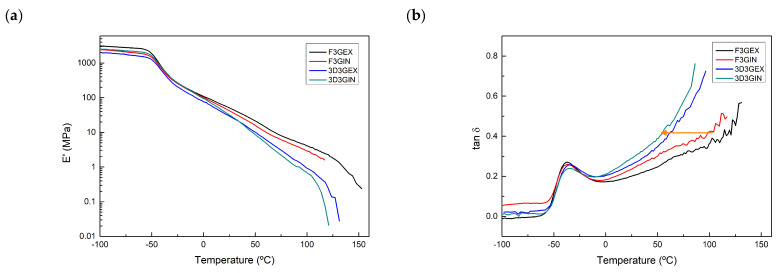
(**a**) Storage modulus and (**b**) tan δ curves of 3D printed specimens.

Regarding DMA analyses of 3D printed specimens, it can be observed that both printed systems show a good thermomechanical behavior (Figure 11). When compared with extruded filaments, a similar behavior is observed. At low temperatures, printed parts show similar modulus values to their corresponding filaments, being the value even slightly higher for 3D3G_IN_. However, at higher temperatures it can be observed that printed specimens showed less stability, since their modulus begins to drop earlier than in the case of the filaments. This lower stability can clearly be seen in tan δ curves, where curves of 3D printed parts tend to infinity at lower temperatures (Figure 11b). This deterioration could be due to further damage suffered by the materials during the high temperature printing process.

When analyzing mechanical properties (Table 3), clear differences in properties can be observed in the printed materials compared with filament systems. Except Young modulus values, other properties show deterioration after the printing process. Both printed parts show higher Young modulus values than their filament counterparts, being it higher for the in-situ prepared one, as observed when studying filament properties. However, stress at yield and stress at break data values of both systems were significantly lower than filaments ones. Strain at break of the printed specimens is also affected and obtained results show less elongation capacity. These poorer mechanical properties shown by the printed parts could be attributed partially to the degradation of the material during the printing process, since temperatures around 200 °C were used. On the other hand, as it could be observed in morphology analyses (Figure 10a,d), the presence of imperfections on the printed parts could also affect the mechanical properties of the systems. The adhesion problems between different layers suggested by SEM analysis could also be responsible for the deteriorated mechanical properties shown by printed parts.

## 4. Conclusions

With the aim to study the possibility of printing a flexible polyurethane filament by FDM with biomedical applicability, a biocompatible waterborne polyurethane was synthesized and filamented. Moreover, and in order to further improved properties and, thus, printability of the systems, cellulose nanofibers and graphene were added as nanoreinforcements. Nanocomposite filaments showed altered properties, which directly related to their printability. It was observed that the addition of graphene resulted in more stable and slightly more rigid filaments, which translated into a better printing process. CNF reinforced filaments showed deteriorations of properties taken place during the printing process. The damaging of the material during the processing counteracted the reinforcement effect cellulose may have supposed, and thus materials with poor mechanical properties and unfitting printability were obtained.

WBPUU/G filaments were successfully printed by FDM and 3D printed parts with good shape fidelity, though slightly damaged properties were obtained. The 3D printed parts showed small imperfection in their morphology and slightly deteriorated thermomechanical and mechanical properties, which could probably be reduced with a further optimization of the printing process. The good printability shown by these nanocomposites suggest potential for their use as more flexible materials in FDM. Furthermore, biocompatibility shown by WBPUU makes it a good candidate for flexible filaments for the biomedical industry.

## Figures and Tables

**Table 1:** 3D Printing parameters.

Extruder Temperature (°C)	200
Bed temperature (°C)	45
Nozzle diameter (mm)	0.6
Printing speed (mm/s)	10
Layer height (mm)	0.2
Infill Pattern	Rectilinear

**Table 2:** Young modulus, stress at yield, stress at break and strain at break values for matrix and composites filaments. Values analyzed using one-way ANOVA with Tukey’s test, different letters indicate statistical differences, where values *p* < 0.05 were considered statistically significant.

Sample	Modulus (MPa)	Stress at Yield (MPa)	Stress at Break (MPa)	Strain at Break (%)
FWBPUU	6.9 ± 1.3 ^a^	1.5 ± 0.1 ^a^	5.4 ± 0.6 ^a^	821.6 ± 35.4 ^a^
F3CNF0_EX_	33.3 ± 5.8 ^b^	5.3 ± 0.3 ^b^	9.1 ± 0.4 ^b^	341.7 ± 53.4 ^b,c^
F3CNF0_IN_	13.7 ± 2.9 ^c^	3.1 ± 0.6 ^c^	4.6 ± 0.8 ^a,c^	427.9 ± 131.0 ^b,d^
F3CNF1_EX_	23.8 ± 7.4 ^d^	3.7 ± 0.3 ^d^	4.6 ± 0.7 ^c^	394.5 ± 62.9 ^b,d^
F3CNF1_IN_	8.6 ± 2.3 ^a^	1.3 ± 0.6 ^a^	2.1 ± 1.2 ^d^	248.5 ± 136.0 ^c,d^
F3G_EX_	32.4 ± 5.6 ^b,d,e^	9.8 ± 0.9 ^e^	28.2 ± 1.8 ^e^	914.8 ± 23.8 ^e^
F3G_IN_	36.4 ± 6.3 ^b,e^	6.4 ± 0.9 ^f^	25.2 ± 0.9 ^f^	995.6 ± 59.7 ^f^

^a,b,c,d,e^ and ^f^ represent different significance groups.

**Table 3:** Young modulus, stress at yield, stress at break and strain at break values for 3D printed specimens.

Sample	Modulo (MPa)	Stress at Yield (MPa)	Stress at Break (MPa)	Strain at Break (%)
3D3G_EX_	30.7 ± 7.8	3.7 ± 0.2	11.4 ± 0.4	467.5 ± 7.3
3D3G_IN_	46.7 ± 8.2	3.7 ± 0.3	12.4 ± 1.3	451.3 ± 29.2

## Data Availability

The data presented in this study are available on request from the corresponding author.
